# Polyaniline enhances the visible light photocatalytic activities of bismuth oxyhalides

**DOI:** 10.1039/d5ra05916j

**Published:** 2025-12-15

**Authors:** Rajesh Kumar, Swadhin Kumar Jena, Million Mulugeta Habtegbrel, Neha Luhakhra, Abhilekha Borah, Aarya Sharma, Prem Felix Siril

**Affiliations:** a School of Chemical Sciences, Indian Institute of Technology Mandi Mandi Himachal Pradesh 175005 India prem@iitmandi.ac.in; b Govt. College Kullu Himachal Pradesh 175101 India

## Abstract

Formation of heterojunction nanocomposites is an effective strategy to improve the photocatalytic activities of semiconducting materials. While, the band structure of the pure materials may guide the design of the heterojunction, the effectiveness of the same will depend on a number of factors such as the type of heterojunction as well as the interfaces. This study investigates the effect of forming a heterojunction between polyaniline (PANI)–and bismuth oxyhalides (BiOX, X = Cl, Br, I) on their photocatalytic performance. The composites were synthesized *via* a simple room-temperature co-precipitation method. Photocatalytic evaluation of orange II (OII) dye as a model pollutant revealed that the PANI/BiOX composites exhibited significantly improved degradation efficiency compared to pristine PANI and bare BiOX counterparts. Notably, the PANI–BiOI (PBI) composite could photocatalytically remove 95.3% of 20 ppm OII dye within 180 min under visible light. PBI could also achieve 90% photoreduction of 100 ppm toxic Cr(vi) to Cr(iii) within 50 min, indicating its superior redox capabilities under visible light. The observed enhancement in photocatalytic activities of the nanocomposites is attributed to synergistic effects including improved light absorption, favorable band edge alignment, and efficient separation of photogenerated charge carriers at the PANI–BiOX interface, thereby reducing recombination losses and enhancing catalytic activity. Reactive species trapping experiments confirmed superoxide radicals (˙O_2_^−^) as the primary oxidative species involved in the dye degradation mechanism. Based on optical and electrochemical findings, formation of a Z-scheme heterojunction is proposed in PBI. This work underscores the potential of PANI–BiOX composites as effective visible-light responsive photocatalysts for environmental cleanup, particularly in the treatment of harmful organic pollutants and heavy metals.

## Abstract 2

Formation of heterojunction nanocomposites is an effective strategy to improve the photocatalytic activities of semiconducting materials.

## Graphical abstract

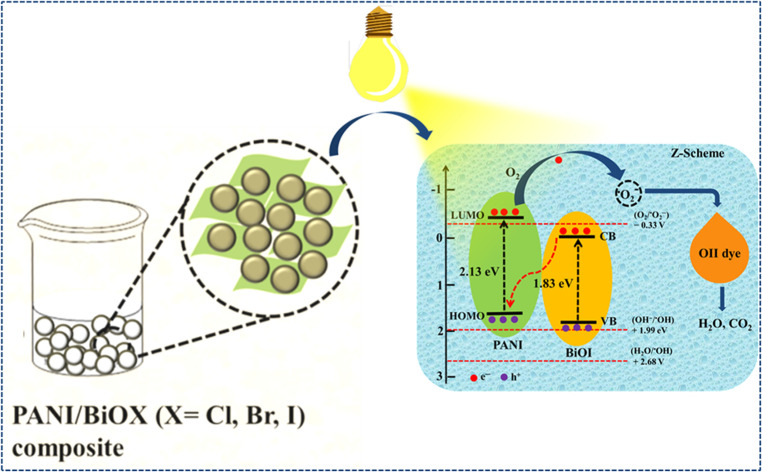

## 1. Introduction

Versatile, visible light active photocatalysts that are capable of promoting a range of oxidation and reduction reactions are required for achieving the sustainability goal of driving chemical reactions using the freely available sun light. Such catalysts should be affordable and their synthesis protocols should be facile so that they may be used widely. Formation of heterojunction nanocomposites is a widely adopted strategy to improve the photocatalytic performance of semiconducting materials.^1,2^ The heterojunction leads to improved absorption of light in a wider range, which is important to capture sunlight.^3^ Moreover, the photogenerated charges can be effectively separated and transferred by the heterojunction. Band edges of the known semiconductors can guide the design of heterojunction.^4^ However, the effectiveness of the heterojunction and hence its photocatalytic activity depends on a range of experimental variables such as the type of heterojunction formed, nature of interaction at the interface, particle size, and nature of surface-active sites. Here, we probe the importance of these factors on the effectiveness of the heterojunction formed between bismuth oxyhalides (BiOX, X = Cl, Br, I) and a conducting polymer, *viz.* polyaniline (PANI).

BiOX emerged as a promising class of photocatalysts, due to their distinctive crystal structures and favorable electronic properties.^5–7^ BiOX crystallize in a tetragonal matlockite-type structure, consisting of alternating [Bi_2_O_2_]^2+^ slabs interleaved with double halide (X^−^) layers.^8^ This layered architecture induces an intrinsic internal electric field, which facilitates the separation of photogenerated charge carriers and enhances photocatalytic performance.^9^ Further, the band gaps of BiOX can be tuned by varying the halide ion in the layered structure. Thus, the band gap for BiOCl, BiOBr and BiOI range from 3.0–3.4, 2.3–2.9, and 1.8–2.1 eV (*versus* NHE), respectively.^10^ As such, BiOBr and BiOI are particularly attractive for visible light driven photocatalysis, enabling them to absorb a significant portion of the solar spectrum. BiOX demonstrated considerable photocatalytic activity in various reactions, including the degradation of organic pollutants, CO_2_ reduction, and water splitting.^11,12^ For example, BiOI exhibited strong visible-light absorption and high photocatalytic efficiency in dye degradation and antibacterial applications.^13^ BiOBr, with its intermediate band gap, balances light absorption and redox potential.^14^

However, heterojunction formation of BiOX with other semiconducting materials, metal oxides and carbon-based materials was found to enhance charge separation, extend light absorption into the visible spectrum, and improve stability.^15^ For example, Ogoh-Orch *et al.* reported the synthesis of BiOI/TiO_2_ heterojunction for the degradation of crude oil hydrocarbons in water.^16^ Marzouqui Al *et al.* synthesized 2D-BiOCl plates with 2D-gCN nanosheets for the photocatalytic degradation of amine-based pharmaceuticals.^17^ Dhillon *et al.* synthesized a multi-functional SnO_2_–BiOBr–rGO heterojunction catalyst with remarkable photocatalytic and electrocatalytic activities.^18^ Ashraf *et al.* synthesized 2D/2D BiOCl/WS_2_ heterojunction for the photcatalytic degradation of malachite green (MG) dye and photoreduction of Cr(vi) using visible light.^19^ In every instance, the composite materials demonstrated enhanced photocatalytic efficiency relative to their individual constituents. Here, we wanted to understand how the nature of the halide (X) in the BiOX affect the photocatalytic activity of a heterojunction composite with PANI. From the reported values of the band structure of the BiOX and PANI, there is a possibility of the formation of a staggered type heterojunction.^20,21^ However, the nature of interaction between BiOX and PANI is difficult to be predicted.

In the recent times, CPs garnered significant interest in the field of photocatalysis owing to their narrow and tunable band gap which make them highly efficient in absorbing UV-vis and NIR radiations.^22,23^ Compared to conventional semiconductors and costly plasmonic photocatalysts, CPs offer a more cost-effective and environmentally friendly alternative. Furthermore, they exhibit excellent photocatalytic activity under natural sunlight, along with efficient charge carrier separation and migration, making them promising candidates for sustainable photocatalytic applications.^24^ PANI, a widely studied CP, gained significant attention in photocatalytic applications due to its extended π-conjugated structure and broad light absorption range spanning the UV-visible to NIR regions.^25,26^ Furthermore, its high specific surface area and charged surface facilitates the adsorption of pollutant molecules, a critical prerequisite for effective photocatalysis.^27^ The limited attempts on the fabrication of PANI–BiOX composites was found to be promising strategy for achieving improved photocatalysis.^28–31^

In this study, we synthesized PANI and BiOX (X = Cl, Br, I) based composite photocatalysts *via* a facile two step co-precipitation method at room temperature. Some guidance was availed from our previous study where we systematically synthesized a series of PANI/BiOBr composites with varying PANI loading of 6 wt% (PB-50), 9.5 wt% (PB-70) and 12 wt% (PB-70). Among these, PB-70 exhibited the highest photocatalytic activity due to the formation of optimal heterojunction between PANI and BiOBr, which facilitated efficient charge separation at the interface.^29^ At lower loadings such as 6 wt%, the conductive network of PANI was insufficient to facilitate efficient interfacial charge transfer, whereas higher PANI loadings (12 wt%) lead to excessive polymer coverage that hindered light absorption and active-site accessibility. To maintain a fair and consistent comparison across the BiOX series, we therefore used the same optimized PANI content (9.5 wt%) to all the samples in present study. The photocatalytic activities of the synthesized composites were studied for the degradation of model pollutant, OII dye under visible light. Furthermore, the versatility of the best photocatalyst was checked for the photoreduction of Cr(vi). Results from the experimental analysis confirmed that the composite formation can effectively enhance the charge carrier transfer at the PANI–BiOX interface. Overall, this work offers significant insights for the development of high performance PANI–BiOX composites for environmental remediation applications.

## 2. Experimental

### 2.1. Chemicals

Aniline (99.5%), ammonium persulphate (APS, (NH_4_)_2_S_2_O_8_, 98%), and bismuth(iii) nitrate pentahydrate (Bi(NO_3_)_3_·5H_2_O, 98%) were acquired from Sisco Research Laboratories Pvt Ltd (SRL). OII dye (≥95% HPLC), potassium bromide (KBr, ≥ 99%), sodium chloride (NaCl, 99%) and *p*-benzoquinone (BQ ≥ 98%) were purchased from Sigma-Aldrich. Sodium chloride (NaCl > 99.5%), potassium iodide (KI > 99.5%) and isopropyl alcohol (IPA > 99.5% HPLC) were purchased from Tokyo Chemical Industry (TCI). Potassium dichromate (K_2_Cr_2_O_7_, 99.9%) and absolute ethanol (99%) were obtained from Merck. Deionized water (DI water, 18.2 MΩ cm) was produced using an ELGA Pure Lab double-stage purification system.

### 2.2. Materials preparation

The nanocomposites were synthesized at the room temperature following a two-step process similar to our previous report.^29^

#### Preparation of PANI

PANI was synthesized *via* a slightly modified chemical oxidative polymerization (COP) method as previously reported.^32^ APS (5.705 g, 0.5 M) was dissolved in DI water (50 mL), while aniline (2.27 mL, 0.5 M) was separately dispersed in DI water (50 mL) at neutral pH. The APS solution was added dropwise to the aniline solution under ice cold conditions. Polymerization commenced within 5 min, as indicated by the appearance of greenish-black precipitates. The reaction mixture was continuously stirred magnetically for 24 h at low temperature (0–6 °C). The resulting polymer was collected and purified by repeated washing with DI water and ethanol, followed by vacuum drying at 70 °C for 8 h.

#### Preparation of PANI–BiOX composites

The PANI–BiOX composites were synthesized *via* a facile co-precipitation method at room temperature. We found from an earlier study on PANI/BiOBr nanocomposites that PANI content of 9.5 wt% was optimum in boosting the catalytic activity of BiOBr. Hence, the PANI content in the composites were kept at 9.5 wt%.^29^ Solution A was prepared by adding Bi(NO_3_)_3_·5H_2_O (810 mg, 0.17 M) to ethanol (10 mL), followed by ultrasonication for 30 min to obtain a homogeneous suspension. Solution B (0.17 M) was separately prepared by dissolving NaCl (99 mg), or KBr (197 mg), or KI (282 mg) in DI water (10 mL). In parallel, solution C was prepared by dispersing PANI (70 mg) in DI water (10 mL), using ultrasonication for 30 min. Subsequently, solution C was gradually introduced into solution A under magnetic stirring at 6000 rpm, followed by the dropwise addition of solution B. The reaction mixture was maintained under magnetic stirring for 24 h at ambient temperature. The resulting solids were collected and washed thrice using ethanol and DI water to remove unreacted residues. The purified composites were then dried in a vacuum oven at 70 °C for 10 h and designated as PBCl, PBBr, and PBI, respectively. For control experiments, pure BiOCl, BiOBr, and BiOI were obtained using the same procedure in the absence of PANI.

### 2.3. Material characterisation

Crystal structure of the synthesized materials was investigated using X-ray diffraction (XRD) analysis performed on a Rigaku SmartLab 9 kW rotating anode diffractometer, operating at 45 kV and 200 mA with Ni-filtered Cu Kα radiation (*λ* = 0.1542 nm). XRD patterns were recorded in the 2*θ* range of 5–90° with a scan rate of 2° min^−1^. The morphology and elemental composition were examined using field emission scanning electron microscope (FESEM, FEI Nova Nano SEM-450) integrated with energy-dispersive X-ray spectroscopy (EDS, EDAX Ametek). High resolution transmission electron microscopy (HRTEM, FEI Tecnai G2 20 S-twin) imaging was carried out at 200 keV accelerating voltage. Fourier transform infrared (FTIR) spectra in the range of 4000–400 cm^−1^ were recorded using Attenuated Total Reflectance FTIR (ATR-FTIR) spectrum 2 instrument. The absorbance spectra in the ultraviolet-visible (UV-vis) range were recorded using a UV 2450 SHIMADZU spectrophotometer, spanning wavelengths from 200 to 800 nm. Diffuse reflectance spectra (DRS) were obtained with a PerkinElmer Lambda 750 UV/Vis/NIR spectrophotometer. Photoluminescence (PL) spectra were measured using a Cary Eclipse fluorescence spectrophotometer (Agilent Technologies), with sample suspensions prepared in ethanol. X-ray photoelectron spectroscopy (XPS) analysis was conducted using a Thermo Scientific NEXSA system with Al Kα radiation (1486.6 eV), and the spectra were deconvoluted using Thermo Scientific's Avantage software.

### 2.4. Evaluation of photocatalytic activities

We studied the photocatalytic decomposition of OII dye using visible light. A homemade photoreactor, equipped with four LEDs (4 × 40 W), was utilized to investigate the photocatalytic reactions. The photocatalyst (5 mg) was added to OII dye solution (10 mL, 20 mg L^−1^) in each experiment. This suspension was stirred in dark for 30 min to achieve the adsorption–desorption equilibrium. The reaction mixture was irradiated with visible light for 180 min. Aliquots (1 mL) were withdrawn after every 30 min, centrifuged at 10 000 rpm to separate the solid catalyst and UV-vis spectra were recorded to monitor the degradation of OII dye. Percentage degradation was calculated by applying the following formula:

1 (1 − *C*_*t*_/*C*_0_) × 100

where *C*_0_ is initial concentration of dye solution and *C*_*t*_ is concentration of dye solution and catalyst after time ‘*t*’ min when exposed to visible light source.

Furthermore, photocatalytic Cr(vi) reduction activity of the best catalyst for the OII dye degradation was also tested. The catalyst (5 mg) was dispersed in Cr(vi) solution (10 mL, 100 ppm), with the pH adjusted to 2.5 using 0.1 M HCl and NaOH. Following the pH adjustment, formic acid (FA, 0.3 mL, 1 M) was added as a hole scavenger, and the suspension was allowed to reach adsorption–desorption equilibrium for 30 min in the dark. The mixture was then subjected to visible light irradiation for 50 min. Aliquots (1 mL) were taken after every 10 min, centrifuged to separate the catalyst, and analyzed using the diphenylcarbazide (DPC) method. To initiate the complex formation, the reaction mixture (0.3 mL) was transferred into a glass test tube (10 mL) containing a mixture of deionized water (2.5 mL), DPC solution (100 µL), and dil. H_3_PO_4_ (60 µL). The resulting solution was thoroughly mixed to ensure homogeneity and then allowed to stand undisturbed for 30 min to facilitate the formation of the characteristic pink-colored DPC–Cr(vi) complex. The pink coloured DPC–Cr(vi) complex was quantified *via* UV-visible spectroscopy to monitor the Cr(vi) reduction. Eqn (1) was used to calculate the percentage reduction.

### 2.5. Photo-electrochemical characterization

Photocurrent response, electrochemical impedance spectroscopy (EIS), and Mott–Schottky (MS) analyses were conducted using a conventional three-electrode setup with aqueous Na_2_SO_4_ (0.1 M) as the electrolyte. The working electrode comprised a fluorine-doped tin oxide (FTO) substrate coated with the catalyst. The catalyst ink was prepared by dispersing the catalyst (5 mg) in a 1 : 1 mixture of the deionized water and isopropyl alcohol (total volume: 480 µL), followed by the addition of Nafion solution (20 µL, 5 wt%). The resulting ink was drop-cast onto the FTO glass, yielding an effective active area of approximately 1.5 cm^2^, and allowed to air dry. Transient photocurrent measurements were performed under visible light irradiation (400–700 nm) from a 40 W LED source placed ∼10 cm from the electrode, with an applied bias of 0.5 V. EIS measurements were carried out at 0.5 V bias in the frequency range of 100 kHz to 0.1 Hz using an AC amplitude of 10 mV. Mott–Schottky analysis were conducted under dark conditions within a potential range of −0.8 to 0.1 V *vs.* NHE keeping a fixed frequency of 1 kHz.

## 3. Results and discussion

The observed results are systematically presented and discussed under different sub headings.

### 3.1. Synthesis and structural studies

**Scheme 1:**
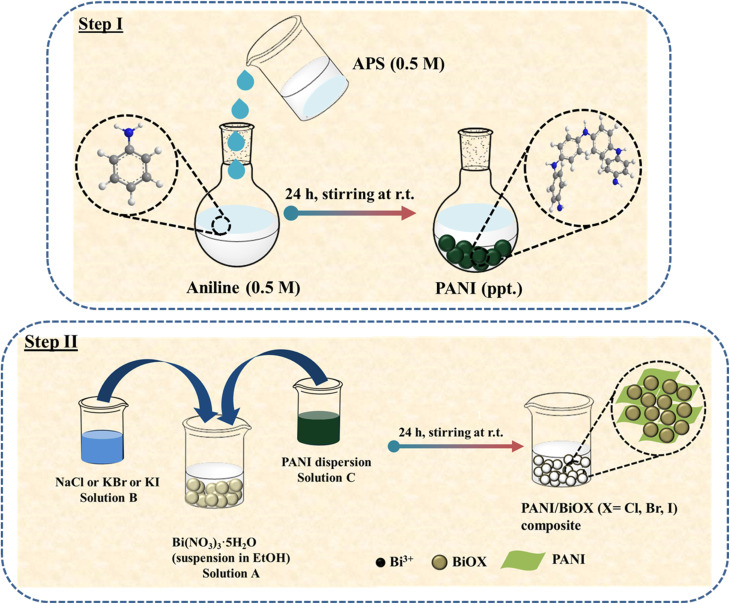
Schematic representation of the synthesis procedure of PANI–BiOX composites.

We used our previously reported synthesis approach, with slight modifications for preparing PANI.^29,32^ PANI with a characteristic greenish-black color was synthesized by the COP of aniline with APS, under ice-cold conditions (∼0–5 °C) (Scheme 1, step 1). The greenish-black color of the resultant PANI indicates the formation of the emeraldine base (EB) form. This form is known to exhibit lower electrical conductivity compared to the emeraldine salt (ES) form, which is typically obtained when the polymerization is carried out in an acidic medium.^33^ Following the synthesis of PANI, PANI–BiOX composites were prepared *via* an *in situ* growth method. Since, heterogeneous nucleation is favoured, the BiOX must have nucleated and grown directly on the surface of the PANI particles in the reaction medium (Scheme 1, step 2). This strategy promotes intimate interfacial contact between BiOX and PANI, potentially enhancing charge carrier separation and transfer dynamics, thereby enhancing photocatalytic performance under visible light. For comparison, pristine BiOX was also synthesized using the same method in the absence of PANI. Pristine BiOCl and BiOBr were obtained as white powders whereas, BiOI was red in color. After the incorporation of greenish-black PANI, the PANI–BiOCl (PBCl) and PANI–BiOBr (PBBr) showed a grey coloration while PANI–BiOI (PBI) was brownish red powder in appearance. Thus, the color difference between the samples further supports the successful composite formation.

**Fig. 1:**
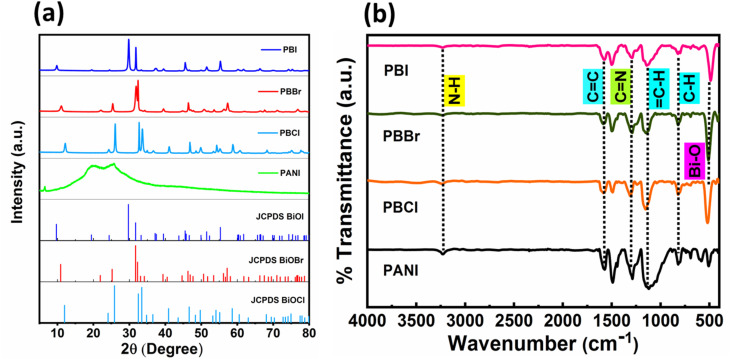
(a) XRD patterns and (b) FTIR spectra of PANI and PANI–BiOX (X = Cl, Br, I) composites.

The solid state of pure PANI, and the corresponding PANI–BiOX composites were analyzed using powder XRD and the results are shown in Fig. 1a. XRD patterns of pure BiOX are shown in SI Fig. S1a. PANI did not show any significant peak due to being amorphous. However, it displayed a broad and diffuse peak centered around 2*θ* ≈ 20–25°. This broad feature arises due to the short-range ordering between PANI chains, particularly in the emeraldine base form.^34^ In contrast, pure BiOX exhibited sharp and well-defined peaks, consistent with the formation of highly crystalline tetragonal BiOX phases. The major diffraction peaks matched closely with those reported in the standard JCPDS files for BiOCl (JCPDS no. 00-090-0393),^35^ BiOBr (JCPDS no. 01-085-0862)^36^ and BiOI (JCPDS no. 01-073-2062),^37^ confirming the successful formation of phase-pure BiOX. The XRD patterns of the PANI–BiOX composites revealed that the major peaks corresponding to BiOX were still clearly present and matched well with their respective reference patterns. This indicates that the BiOX crystal structure remained intact upon nanocomposite formation with PANI. However, the characteristic broad peak of amorphous PANI was not distinctly visible in the composites due to its diffuse XRD pattern and lesser amount, which can be easily masked by the intense peaks of the predominant BiOX.

FTIR spectroscopy was employed to investigate the functional groups in pure PANI and PANI–BiOX composites (Fig. 1b and SI Fig. S1b). The spectra reveal key vibrational features that confirm the formation of PANI and its successful integration with BiOX. In the FTIR spectrum of bare PANI, prominent peaks were observed at 1567 and 1486 cm^−1^, which correspond to the C=C stretching vibrations of the quinonoid and benzenoid units, respectively.^38^ These peaks are characteristic of the EB form of PANI, reflecting its mixed oxidation state and conjugated structure. The absorption band at 1303 cm^−1^ is assigned to the C=N stretching vibration of the imine group.^39^ This peak was also observed in the spectra of all PANI–BiOX composites, indicating the retention of the chemical integrity of PANI even after composite formation. Another important vibrational feature appears at 1128 cm^−1^, corresponding to the in-plane deformation (bending) of the =C–H bond in the aromatic structure. Notably, this band exhibited a slight shift to higher wavenumbers in the PANI–BiOX composites, suggesting a possible electrostatic interactions or weak hydrogen bonding between the PANI chains and the surface of BiOX nanoparticles. An additional peak at 820 cm^−1^ is due to out-of-plane C–H bending vibrations in a 1,4-disubstituted aromatic ring.^34^ The distinctive Bi–O stretching vibration for BiOX was observed at around 510 cm^−1^. This band was consistently present in all the PANI–BiOX composites while being absent in PANI, reaffirming the presence of BiOX within the composite matrix.^40–42^

### 3.2. Photocatalytic activity

**Fig. 2:**
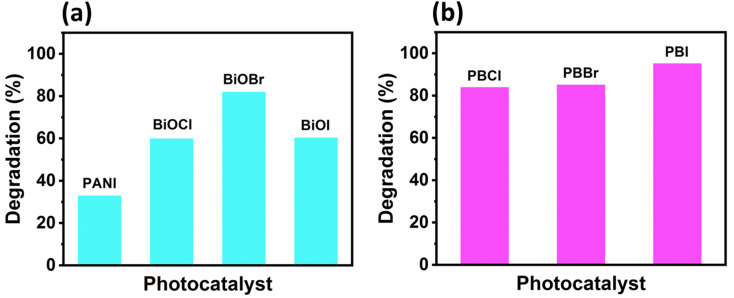
Photocatalytic removal percentage of OII dye under visible light irradiation using: (a) bare PANI and BiOX as catalysts and (b) different PANI–BiOX composites as catalysts.

The photocatalytic behaviors of PANI, BiOX, and PANI–BiOX composites were systematically evaluated using OII dye as a model organic pollutant. Adsorption of OII under the dark for 30 min followed by the photocatalytic degradation was monitored over a period of 180 min using UV-visible spectroscopy. The time-resolved UV-visible absorbance spectra showing the photodegradation of OII dye using pristine catalysts and PANI–BiOX composites are presented in SI Fig. S2 and S3, respectively. A progressive decrease in the intensity of the characteristic absorption band of OII at 483 nm was observed. Simultaneous decrease of the intensity of the minor peak at ∼300 nm was also observed. Presence of PANI in the nanocomposite was found to enhance the surface adsorption properties of the resulting PANI–BiOX composites. This may be due to the increased surface area and improved interfacial interactions provided by PANI.^43^ Pure PANI showed significantly high ability to adsorb the OII dye. Since the nanocomposite contained 9 wt% PANI, the quantity of pure PANI present was proportionally adjusted and calculated to be approximately 0.45 mg in the 10 mL reaction system. A comparison of the maximum photocatalytic removal% by the bare materials after 180 min irradiation are shown in Fig. 2a whereas the data for the nanocomposites is shown in Fig. 2b. Bare PANI could only remove 34% of the OII dye in 180 min whereas, the BiOX showed much higher removal. Among the pure BiOX, BiOBr exhibited the highest photocatalytic performance, achieving approximately 82% removal of OII in 180 min. BiOCl and BiOI showed similar photocatalytic activities (∼62% removal) which was lower than BiOBr.

In contrast to the bare photocatalysts, the PANI–BiOX composites exhibited a pronounced enhancement in photocatalytic performance as shown in Fig. 2b. PBI exhibited the highest removal efficiency, achieving approximately 95.3% removal of OII dye, followed by PBBr (85.2%) and PBCl (84%) within the same irradiation period. The quantum of enhancement in photocatalytic removal% of the nanocomposites when compared to the pure BiOX followed the order PBI > PBCl > PBBr. These results clearly indicate that PBI possesses enhanced photocatalytic performance in comparison to the other composites and pristine materials.

**Fig. 3:**
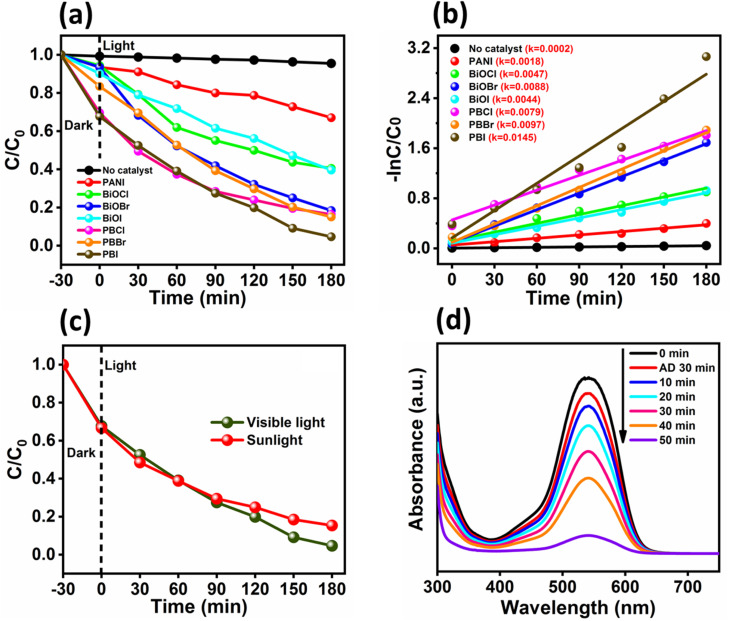
(a) Decrease in the concentration of OII dye (20 ppm, 10 mL) with time in the presence of photocatalysts (5 mg), initially due to adsorption (up to 30 min) and then due to photocatalytic degradation. (b) Linear plots showing the pseudo-first order model kinetic model for the OII dye photodegradation. (c) Comparison of the photocatalytic activity of the best-performing catalyst (PBI) for the degradation of OII dye under both visible light and natural sunlight. (d) UV-vis spectra showing the photocatalytic reduction of Cr(vi) (100 ppm, 10 mL) using visible light using PBI as the catalyst.

Furthermore, the photocatalytic degradation kinetics of the OII dye was investigated by applying a pseudo-first-order reaction model to the time-dependent concentration data obtained from UV-visible spectra. The kinetic behavior was analyzed using the integrated rate law expressed as:^44^ln(*C*/*C*_0_) = −*kt*where, *C*_0_ represents the initial dye concentration at time *t* = 0, *C* is the concentration at a given time *t*, and *k* is the apparent first-order rate constant (min^−1^). Kinetic plots are shown in Fig. 3. Rate constants for the OII dye degradation in presence of the catalysts followed the order: PBI (0.0145 min^−1^) > PBBr (0.0097 min^−1^) > BiOBr (0.0088) > PBCl (0.0079 min^−1^) > BiOCl (0.0047) > BiOI (0.0044) > PANI (0.0018). The highest rate constant observed for the PBI composite confirms its superior photocatalytic activity. The photocatalytic activity of the bare BiOX increased invariably on the nanocomposite formation. But, the quantum of enhancement is not to the same extent, although the quantity of PANI in the nanocomposite was the same. While, the rate constant for OII degradation increased by 230% for BiOI with the nanocomposite formation with PANI, the increment was a mere 10% for BiOBr and 68% for BiOCl.

To further evaluate the practical applicability of the best-performing catalyst (PBI), we conducted a comparative study of its performance for the photocatalytic degradation of OII dye under both visible light and natural sunlight, as shown in Fig. 3c. PBI could achieve 95.3% dye removal under visible light within 180 min. Almost the similar performance was observed under sunlight exposure, where about 85% dye removal could be achieved. The slight difference in the photocatalytic activity under sunlight can be attributed to variation in sunlight intensity and spectral distribution with the white light source (LED). These results illustrate the remarkable potential of PBI as a visible light active photocatalyst, with promising applicability for both artificial and natural light-driven environmental remediation and photocatalysis.

Beyond dye degradation, the PBI was further evaluated for its versatility by applying it to the photocatalytic reduction of Cr(vi) to less harmful Cr(iii) (Fig. 3d). In this case, we utilized an optimized experimental procedure with slight modifications.^45^ The composite demonstrated outstanding performance, achieving a 90% reduction of Cr(vi) within 50 min under visible light. This high reduction efficiency is attributed to the synergistic effect of the BiOI semiconductor and the conductive PANI matrix, which enhances charge separation and transfer, thereby facilitating more effective redox reactions. The efficient reduction of Cr(vi) highlights the potential of PBI as a multifunctional photo-redox catalyst for environmental mitigation applications beyond organic dye degradation. Furthermore, we compared the photocatalytic degradation performance of BiOI based composites from the literature for various pollutants with PBI and the results are shown in SI Table S1. Evidently, PBI outperformed most other heterojunction nanocomposites of BiOI, as it could achieve high dye removal in shorter time with lower amount of the catalyst, even under less intense light.

**Fig. 4:**
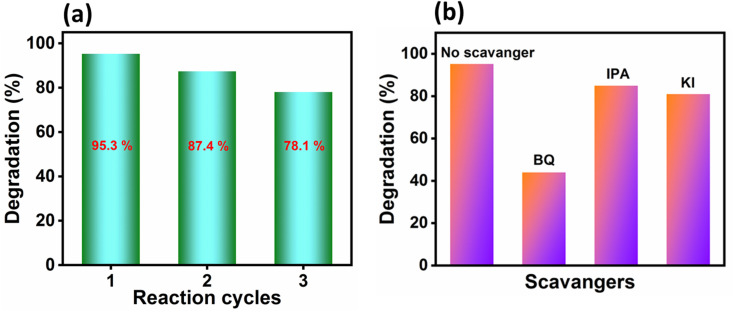
(a) Photocatalytic removal percentage of OII dye for three reaction cycles and (b) scavenger study results using PBI composite under visible light.

The recyclability of PBI was evaluated over three consecutive photocatalytic degradation cycles and the results are shown in Fig. 4a. The catalyst was recovered *via* centrifugation after each reaction cycle, thoroughly washed with deionized (DI) water and ethanol for reused in the following cycle. The photocatalytic performance was monitored based on the dye removal efficiency of OII dye, which remained at approximately 78.1% after the third cycle. While the PBI composite retained a considerable portion of its activity, a gradual decline in photocatalytic efficiency was apparent. This was primarily due to the physical loss of catalyst during the recovery and washing steps rather than to any intrinsic changes in the catalyst itself.

To find out the dominant role of reactive oxygen species (ROS) involved in the photocatalytic degradation mechanism of OII dye using the PBI composite, a series of scavenger experiments were performed (Fig. 4b).^46^ Specific quenchers were employed to selectively inhibit key reactive species: BQ (1 mM) for superoxide radicals (˙O_2_^−^), IPA (1 mM) for hydroxyl radicals (˙OH), and KI (1 mM) for the photogenerated holes (h^+^). Each scavenger was introduced into the reaction system immediately prior to the addition of the photocatalyst, while all other reaction conditions, including catalyst dosage (5 mg) and dye concentration (20 ppm), were held constant to ensure comparability. The results of scavenger study in Fig. 4b suggest minimum degradation in the presence of BQ (44%), followed by KI (82%) and IPA (84%). Thus, ˙O_2_^−^ radicals play a pivotal role in the photocatalytic process with minor contributions from ˙OH and h^+^. To further investigate the major role played by ˙O_2_^−^, we employed nitroblue tetrazolium (NBT, 100 µM) as a probe to quantify its concentration.^47,48^ A progressive decease in the absorbance intensity of NBT at *λ* = 259 nm was observed with time as shown in SI Fig. S4, indicating the formation of ˙O_2_^−^ radical. The ˙O_2_^−^ radicals were generated with a rate constant of 0.0131 min^−1^ and its concentration was estimated to be around 2.62 × 10^−5^ M after 50 min of irradiation in presence of PBI (SI Fig. S7).

Further, we evaluated the effect of varying pH during the photocatalytic OII dye removal and the reduction of hexavalent Cr. Apart from the neutral condition, acidic condition was achieved by adding HCl (1 mL of 0.1 M, pH = 2–3) to the reaction mixture before exposing to visible light, keeping all other reaction parameters same. Similarly, basic condition was achieved by adding NaOH (1 mL of 0.1 M, pH = 9–10) to reaction mixture. Photocatalytic degradation of OII dye was found to be strongly pH dependent, showing the highest activity at neutral pH compared to acidic and basic conditions (Fig. S5a). This behavior can be attributed to the interplay between surface charge, ˙O_2_^−^ radical generation and the protonation state of PANI.^49^ A much-detailed study is required to unearth the role of each parameter, which is beyond the scope of the present study. The pH of the reaction medium also was found to affect the photoreduction of hexavalent Cr(vi) as shown in Fig. S5b. Photoreduction of Cr(vi) is favoured under acidic pH, as reported in earlier studies. Effect of pH on the photoreduction of Cr(vi) is typically attributed to the variation in concentration of various chromate species having difference in their reduction potentials.^50^ However, the role of the change in conductivity and surface charge of PANI also cannot be ruled out.

### 3.3. Morphological, compositional and optical analysis

**Fig. 5:**
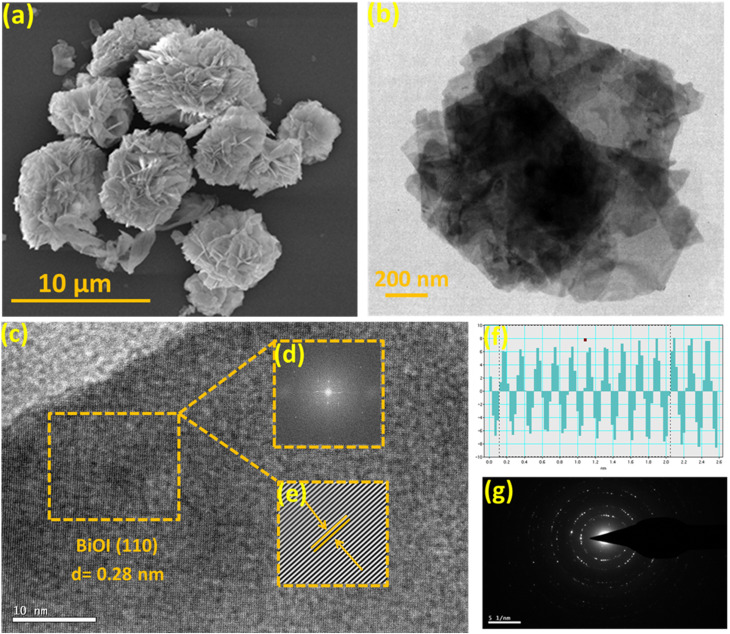
Characteristics of the PBI composite: (a) FESEM image (b and c) HRTEM images, (d and e) FFT and IFFT images of selected area showing uniform fringes, (f) line analysis showing uniform width of fringes in the selected area, and (g) SAED pattern.

The microstructural characteristics of the PANI, BiOI and the representative composite PBI were further investigated using FESEM. The FESEM images revealed a plate-like morphology for pure BiOI (SI Fig. S6a). In contrast, pure PANI displayed an aggregated fibrous microstructure, suggesting the formation of intertwined polymer chains (SI Fig. S6b). Significant morphological transformation was observed on composite formation, where the PBI showed a distinctive flower-like architecture as evident in Fig. 5a. Furthermore, we performed HRTEM, FFT and IFFT analysis of PBI and the results are presented in Fig. 5c–f. The HRTEM image in Fig. 5c revealed well-defined lattice fringes with an interplanar spacing of approximately 0.28 nm, which can be indexed to the (110) crystallographic plane of BiOI.^51^ A specific region from Fig. 5c was selected for FFT analysis to evaluate the periodicity and crystallinity of the lattice structure (Fig. 5d), followed by the generation of the IFFT image (Fig. 5e) to isolate the real-space lattice information. The IFFT image exhibited uniform and continuous lattice fringes, with no discernible structural defects, indicating high crystallinity in the BiOI domains. Moreover, line profile analysis (Fig. 5f) of the selected region demonstrated consistent lattice fringe spacing, further validating the structural uniformity of the material. Additionally, SAED pattern (Fig. 5g) displayed concentric diffraction rings, confirming the polycrystalline nature of the PBI composite. However, there were domains in HRTEM images of the PBI composite with no lattice fringes, because of the amorphous nature of PANI.

**Fig. 6:**
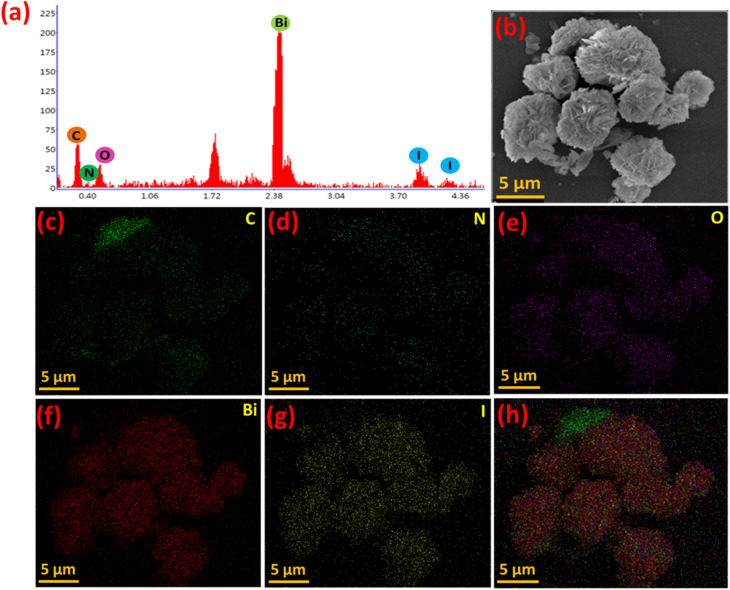
(a) EDX spectrum, (b) FESEM image, and (c–h) elemental maps of the PBI composite.

Furthermore, the EDX attached to FESEM confirmed the presence of all the expected elements (C, N, O, Bi, I) in the representative composite, PBI as shown in Fig. 6a. The FESEM image of PBI is presented in Fig. 4b and the corresponding elemental maps for C, N, O, Bi and I are shown in Fig. 6c–h. The elemental mapping revealed that Bi, O, and I were uniformly distributed across the sample, consistent with the predominant presence of BiOI in the composite matrix. In contrast, the characteristics signals for C and N in PANI moiety were less intense, but present throughout, reflecting the comparatively lower loading of PANI within the hybrid structure. The spatial correlation observed in the elemental mapping strongly supports the successful synthesis of the PBI composite. The distribution patterns indicate that PANI domains are mostly embedded within the BiOI matrix, implying intimate interfacial contact between the two phases which is essential for the charge carrier separation and transport in photocatalytic applications. Albeit, there were some domains enriched PANI phases were also observed.

**Fig. 7:**
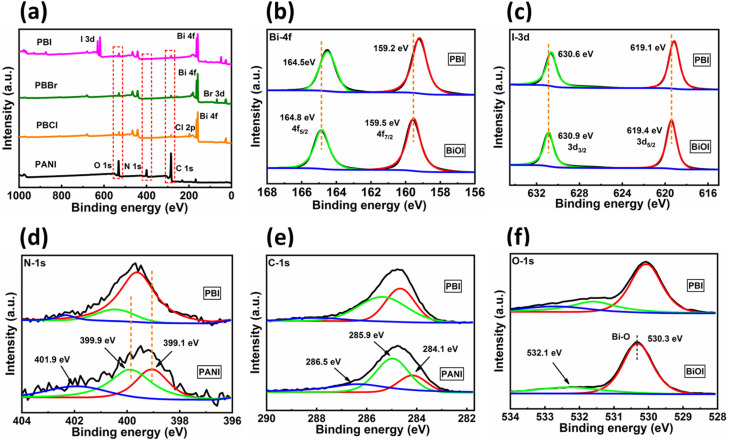
(a) XPS survey spectra of PANI and as synthesized composites. XPS deconvoluted spectra for (b) Bi-4f, (c) I-3d, (d) N-1s, (e) C-1s, and (f) O-1s.

XPS analysis was performed to investigate the elements present and valence states in all the composites. The survey spectra for bare PANI and BiOX are provided in SI Fig. S7. The survey spectra of the nanocomposites along with bare PANI is shown in Fig. 7a. Presence of C, N, O, Bi and halogens, X (X = Cl, Br, I) are present in the PANI–BiOX composites. The narrow scan spectra of PANI, BiOI and a representative composite, PBI are presented in Fig. 7b–f. The high resolution XPS spectrum of bare BiOI shows two strong peaks corresponding to the Bi 4f_7/2_ and Bi 4f_5/2_ at 159.5 and 164.8 eV, respectively.^52^ Upon formation of the PBI composite, these peaks shifts toward lower binding energy at 159.2 and 164.5 eV, respectively. The narrow scan XPS spectrum of bare BiOI exhibits two distinct peaks at 619.4 and 630.9 eV, corresponding to the I 3d_5/2_ and I 3d_3/2_, respectively.^53^ After forming the PBI, each of these peaks shifts towards lower binding energy at 619.1 and 630.6 eV, respectively, which are attributed to the presence of iodide ions in different electronic environment. The narrow scan of N 1s in PANI shows three peaks at 399.1, 399.9, and 401.9 eV, corresponding to amine nitrogen (–NH–), imine nitrogen (=N–), and protonated nitrogen species, respectively.^31,54^ All the N 1s peaks were shifted towards higher binding energies in PBI, indicating strong interfacial electronic interaction and charge redistribution in the composite. The high-resolution C 1s spectra of PANI shows three peaks at 284.1, 285.9, and 286.5 eV, which are attributed to C–C, C=C, and C–O bonds, respectively.^55^ Similar peaks with slight peak shift towards higher binding energy were observed in PBI composite, indicating chemical interaction at the interface. The deconvoluted spectra of O 1s in BiOI exhibits two peaks at 530.3 and 532.1 eV which are assigned to Bi–O lattice oxygen and adsorbed oxygen species, respectively.^56,57^ These peaks were present in the composite PBI at slightly higher binding energy, confirming the interfacial interaction between PANI and BiOI.

**Fig. 8:**
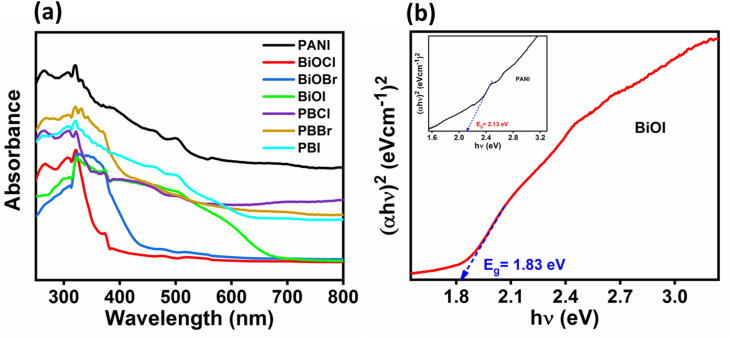
(a) Solid state DRS spectra of synthesized samples, (b) Tauc plot showing band gap of PANI (inset) and BiOI.

DRS analysis was performed for all the synthesized samples and the corresponding spectra are shown in Fig. 8a. PANI showed characteristic absorbance in the UV-vis-NIR regions. However, the pristine BiOCl and BiOBr samples exhibit strong absorption predominantly in the UV region, whereas BiOI shows an extended absorption in the visible region also, starting from the UV, with absorption edge around 600 nm. Upon the formation of the nanocomposites, a significant enhancement in light absorption is observed in the visible region extending well into the NIR region. This indicates a synergistic effect between PANI and the BiOX in broadening the optical response of the composites. To determine the optical bandgap energies, the Kubelka–Munk function was employed using the relation:^58^*α*ℏ*ν* = *A*(ℏ*ν* − *E*_g_)^*n*^where ‘*α*’ is the absorption coefficient, ‘ℏ’ is Planck's constant, ‘*ν*’ is the frequency of the incident light, ‘*A*’ is a material-dependent constant, and ‘*E*_g_’ is the optical bandgap energy. The exponent ‘*n*’ corresponds to the nature of the electronic transition. The calculated bandgap energies were found to be 1.83 eV for pristine BiOI and 2.13 eV for pure PANI (Fig. 5b). Further, the band gaps for bare BiOCl and BiOBr are 3.38 and 2.85 eV, respectively (SI Fig. S8).

### 3.4. Photo-electrochemical studies

**Fig. 9:**
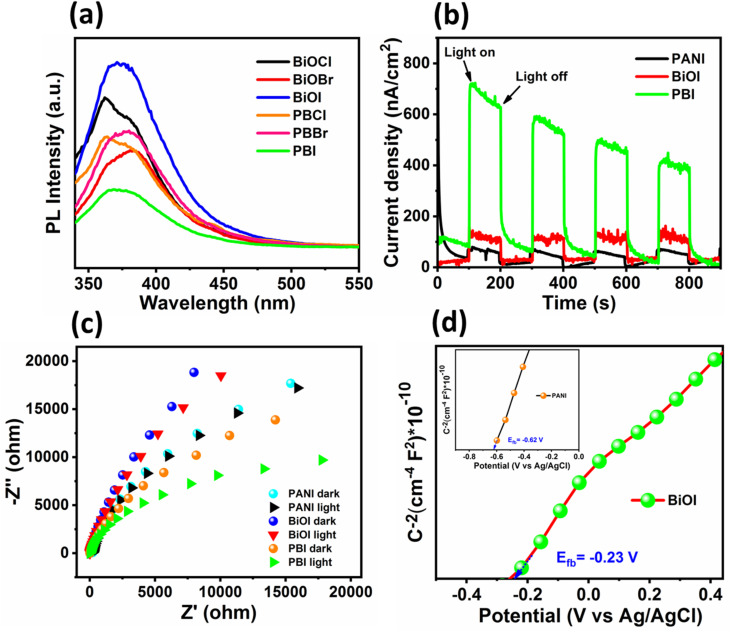
(a) PL spectra of synthesized materials. (b) Photocurrent response and (c) EIS plots of PANI, BiOI and PBI. (d) Mott–Schottky curves of BiOI and PANI.

The superior photocatalytic activity observed in the PBI composite may be attributed to the enhanced absorption of light, generation, separation, and transport of photogenerated electron–hole pairs. We carried out optical and electrochemical analysis to validate this. PL analysis in Fig. 9a depicts significant reduction in intensity of all the PANI–BiOX composites when compared to their pristine BiOX counterparts, indicating suppressed radiative recombination of electron–hole pairs upon incorporation of PANI. Notably, the PBI demonstrated the lowest PL intensity among all the tested samples. Moreover, the fluorescence intensity was the highest for BiOI which was suppressed to the maximum extent by the heterojunction formation. This pronounced quenching in PL intensity implies that PANI facilitates efficient charge carrier separation, minimizing recombination losses which can result in improved photocatalytic activity under visible light irradiation.^59^ BiOBr showed lower PL intensity than BiOCl and BiOI. This explains the observed higher photocatalytic activity of BiOBr among the pure BiOX. Moreover, the PL intensity was the highest for BiOI. High rate of recombination of charge carriers in BiOI happens due to its narrow band gap, weak internal electric field and stronger polaronic effects, which is already reported in the literature.^60,61^

To elucidate the efficacy of photogenerated charge carrier separation and transport in the PBI composite, photocurrent measurements were also conducted. As shown in Fig. 9b, the PBI composite demonstrated higher photocurrent response relative to bare BiOI and PANI, indicating more efficient generation and separation of electron–hole pairs in the composite system.^62^ To further evaluate the charge transfer dynamics, EIS was performed under dark and illuminated conditions for PANI, BiOI, and the PBI composite. The Nyquist plots in Fig. 9c revealed that the PBI composite showed smaller semicircular arc radius compared to pure BiOI and PANI. This reduction in resistance to charge transfer is indicative of enhanced electrical conductivity and charge mobility in the composite.^63^

To further investigate the electronic properties of PANI and BiOI, Mott–Schottky (M–S) analysis was carried out at a frequency of 1000 Hz (Fig. 9d). This technique was employed to determine the flat-band potentials and to deduce the semiconductor type of the individual components.^64,65^ The positive slopes observed in the M–S plots for both PANI and BiOI confirm their n-type semiconductor behavior, as per the classical interpretation where a positive slope indicates an n-type and a negative slope indicates a p-type semiconductor.^66^ From the intercepts of the M–S plots, the flat-band potentials (*E*_fb_) of PANI and BiOI were estimated to be −0.62 V and −0.23 V *versus* the Ag/AgCl reference electrode, respectively. For n-type semiconductors, the energy difference between the conduction band minima and the *E*_f_ is 0.2 eV.^67^ Thus, the conduction band positions of PANI and BiOI were found to be −0.82 V & −0.43 V, respectively. These values were subsequently converted to the normal hydrogen electrode (NHE) scale using the standard conversion formula: *E*_NHE_ = *E*_Ag/AgCl_ + 0.197 V. Now, the conduction band potentials were computed to be −0.623 V and −0.233 V for PANI and BiOI, respectively. Considering the optical band gap of the samples, the valence band (VB) edge positions of PANI and BiOI were calculated using the relation *E*_VB_ = *E*_g_ + *E*_CB_, which was approximately +1.507 V and +1.597 V *vs.* NHE, respectively.

### 3.5. Comparison of physical properties

The above presented opto-electric properties are helpful in interpreting the observed trends in catalytic activities. A summary of the relevant properties is given in Table 1.

**Table 1:** Summary of the band gap, PL intensity, photocatalytic performance (% removal and rate constants for OII dye degradation)

Sample	Band gap/eV	PL intensity	Rate constant/min^−1^	% Removal
PANI	2.13	—	0.0018	34
BiOCl	3.38	0.786	0.0047	62
BiOBr	2.85	0.517	0.0088	82
BiOI	1.83	0.967	0.0044	62
PBCl	—	0.583	0.0079	84
PBBr	—	0.618	0.0097	85.2
PBI	—	0.314	0.0145	95.3

Among the BiOX, the photocatalytic activity was the highest for BiOBr because of its intermediate band gap allowing it to absorb visible light along with relatively lower recombination of its charge carriers, as evident from its low PL intensity. The catalytic activity of BiOCl was lower than BiOBr because of its inability to generate charge carriers in visible light, as its band gap lies in UV region. On the other hand, BiOI showed a high rate of recombination of its charge carriers, as evident from its intense PL, limiting its photocatalytic activity. However, significant improvement in its photocatalytic activity was evident on the nanocomposite formation. This must be due to the efficient transfer of its charge carriers because of the heterojunction formed. PBI showed much lower PL intensity and a high photocurrent than BiOI and PANI. Of course, the ability of PANI adsorb the OII dye also is one of the reasons for the observed improved photocatalytic activity of the nanocomposites.

### 3.6. Photocatalytic activity mechanism

**Scheme 2:**
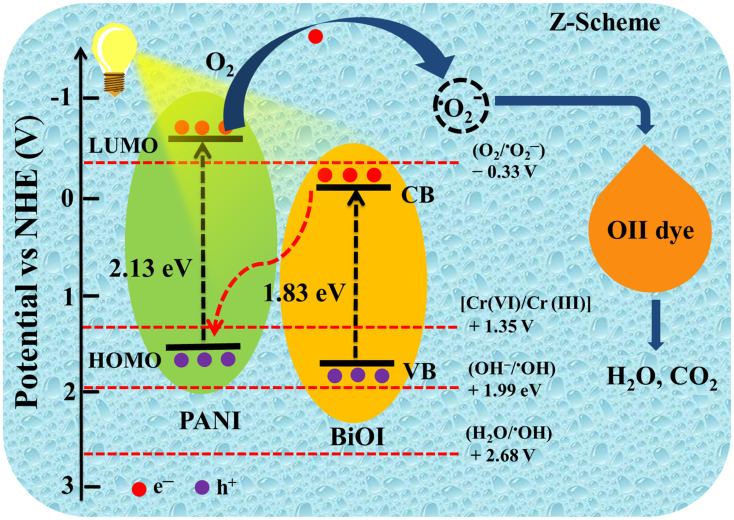
Proposed mechanism for the photocatalytic degradation of OII dye under visible light over the PBI composite.

Based on our findings from radical scavenger experiments, combined with optical absorption and electrochemical analysis, we propose the formation of a direct Z-scheme heterojunction in the PBI composite (Scheme 2). Upon irradiation with visible light, both PANI and BiOI are photoexcited, generating electron–hole pairs. Photoexcited electrons are promoted to LUMO in PANI from the HOMO, while electrons are excited from VB to CB in BiOI. However, the CB potential of BiOI (−0.233 V *vs.* NHE) is thermodynamically insufficient to reduce O_2_ to ˙O_2_^−^ radical, which requires a more negative potential (−0.33 V *vs.* NHE).^68^ Despite this limitation, the radical scavenging studies indicate that ˙O_2_^−^ is the major ROS responsible for the photocatalytic activity of PBI. This contradiction suggests that another pathway must be involved in the generation of these radicals. To validate these findings, a direct Z-scheme heterojunction mechanism is proposed. In this charge transfer pathway, the photogenerated electrons in the CB of BiOI recombine with holes in the HOMO of PANI. This recombination preserves the electrons with strong reduction abilities in the LUMO of PANI and holes with high oxidation ability the VB of BiOI, respectively. The electrons in the LUMO of PANI are now thermodynamically capable of reducing O_2_ to ˙O_2_^−^ radicals due to more negative potential (−0.62 V) than −0.33 V. Thus, this type of charge transfer mechanism explains the experimental observation of efficient ˙O_2_^−^ generation. Further, the more negative potential of the nanocomposite is also beneficial in effectively reducing the toxic Cr(vi) to less toxic Cr(iii). Thus, the present study highlights the synergistic charge separation and redox capability of the PBI system, making it an effective photocatalyst for the environmental remediation applications using visible light.

## 4. Conclusions

In this comparative investigation, three distinct polyaniline/bismuth oxyhalide (PANI–BiOX; X = Cl, Br, I) composites were successfully synthesized *via* a facile room-temperature chemical route. The photocatalytic activity of the composites and the pristine materials (PANI and BiOX) was systematically evaluated by monitoring the degradation of orange II (OII) dye under visible light irradiation. The enhanced photocatalytic activity observed in the PANI–BiOX composites is attributed to the improved optical and electrochemical performances. Photoluminescence (PL) analysis proves that the synergistic interaction between PANI and the BiOX facilitates the efficient photogeneration of charge carriers while significantly suppressing their recombination. Among the synthesized composites, PANI–BiOI (PBI) exhibited the highest photocatalytic performance for degrading OII dye and showed versatility for the photocatalytic reduction of harmful Cr(vi) under visible light. The improved photocatalytic performance is attributed to the well-matched conduction and valence band edge potentials of BiOI and PANI, which facilitate the formation of a direct z-scheme type heterojunction leading to efficient charge carrier separation and interfacial charge transfer. Radical scavenging experiments indicated that the superoxide radicals (˙O_2_^−^) were the main reactive oxygen species responsible for degrading pollutants. These findings underscore the potential of PANI–BiOX hybrid systems as promising visible light responsive photocatalysts for the removal of organic and inorganic pollutants in wastewater. Beyond dye degradation, these composites can be explored for broader applications, including degradation of emerging pharmaceuticals contaminants, CO_2_ reduction, and antibacterial surfaces.

## Conflicts of interest

There are no conflicts of interests to declare.

## Supplementary Material

RA-015-D5RA05916J-s001

## Data Availability

All the data generated related to this paper are presented in the manuscript or the supplementary information (SI). Supplementary information: XRD pattern, and FTIR spectra of as synthesized BiOX, UV-vis absorbance spectra of photocatalytic OII dye degradation under visible light for PANI, BiOCl, BiOBr, and BiOI photocatalysts, UV-vis absorbance spectra of photocatalytic OII dye degradation under visible light for PBCl, PBBr, and PBI composite photocatalysts, Time-resolved UV-visible absorbance spectra of NBT during visible light irradiation in presence of PBI composite and the respective kinetic curve, Curves showing the effect of varying pH (acidic, 0.1 M HCl neutral and basic, 0.1 M NaOH) on the photocatalytic performance of PBI on the degradation of OII dye, and the photocatalytic reduction of Cr(vi) to Cr(iii), FESEM images of BiOI and PANI, XPS survey spectrum of PANI and as synthesized nanocomposites, Tauc plot showing band gap enegy of BiOCl and BiOBr and a table showing comparison of the photocatalytic activity of the PANI-BiOI with some other hybrid composites for the degradation of various pollutants under visible light. See DOI: https://doi.org/10.1039/d5ra05916j.
