# Supplementary material for: Polyaniline enhances the visible light photocatalytic activities of bismuth oxyhalides
Source: RSC Adv. 2025 Dec 15;15(58):50127–41. doi: 10.1039/d5ra05916j (PMC12705227; doi:10.1039/d5ra05916j)
Supplement: RA-015-D5RA05916J-s001 [file RA-015-D5RA05916J-s001.pdf]

Supplementary Information (SI) for RSC Advances.
This journal is © The Royal Society of Chemistry 2025

Polyaniline enhances the visible light photocatalytic activities of bismuth oxyhalides

Rajesh Kumar[a,b], Swadhin Kumar Jena[a], Million Mulugeta Habtegbrel[a], Neha Luhakra[a], Abhilekha Borah[a], Aarya Sharma[a] and Prem Felix Siril[a]

[a] School of Chemical Sciences, Indian Institute of Technology Mandi, Mandi, Himachal Pradesh-175005, India
[b] Govt. College Kullu, Himachal Pradesh- 175101, India
*Corresponding author: prem@iitmandi.ac.in

Table of content:

Sr No.	Contents	Pages
1.	Fig. S1 XRD pattern (a), and FTIR spectra (b), of as synthesized BiOX	2
2.	Fig. S2 UV-vis absorbance spectra of photocatalytic OII dye degradation under visible light for : (a) PANI, (b) BiOCl, (c) BiOBr, and (d) BiOI photocatalyst	3
3.	Fig. S3 UV-vis absorbance spectra of photocatalytic OII dye degradation under visible light for : (a) PBCl, (b) PBBr, and (c) PBI composite photocatalyst	4
4.	Fig. S4 (a) Time-resolved UV-visible absorbance spectra of NBT during visible light irradiation in presence of PBI composite and (b) the respective kinetic curve.	5
5.	Fig. S5 Effect of varying pH (acidic, 0.1 M HCl neutral and basic, 0.1 M NaOH) on the photocatalytic performance of PBI on **(a)** degradation of OII dye, and **(b)** photocatalytic reduction of Cr(VI) to Cr(III).	6
6.	Fig. S6 FESEM images of (a) BiOI and (b) PANI	6
7.	Fig. S7 XPS survey spectrum of PANI and as synthesized nanocomposites	7
8.	Fig. S8 Tauc plot showing band gap enegy of (a) BiOCl and (b) PANI BiOBr	7
9.	Table: S1 Comparison of the photocatalytic activity of the PANI-BiOI with some other hybrid composites for the degradation of various pollutants under visible light	8

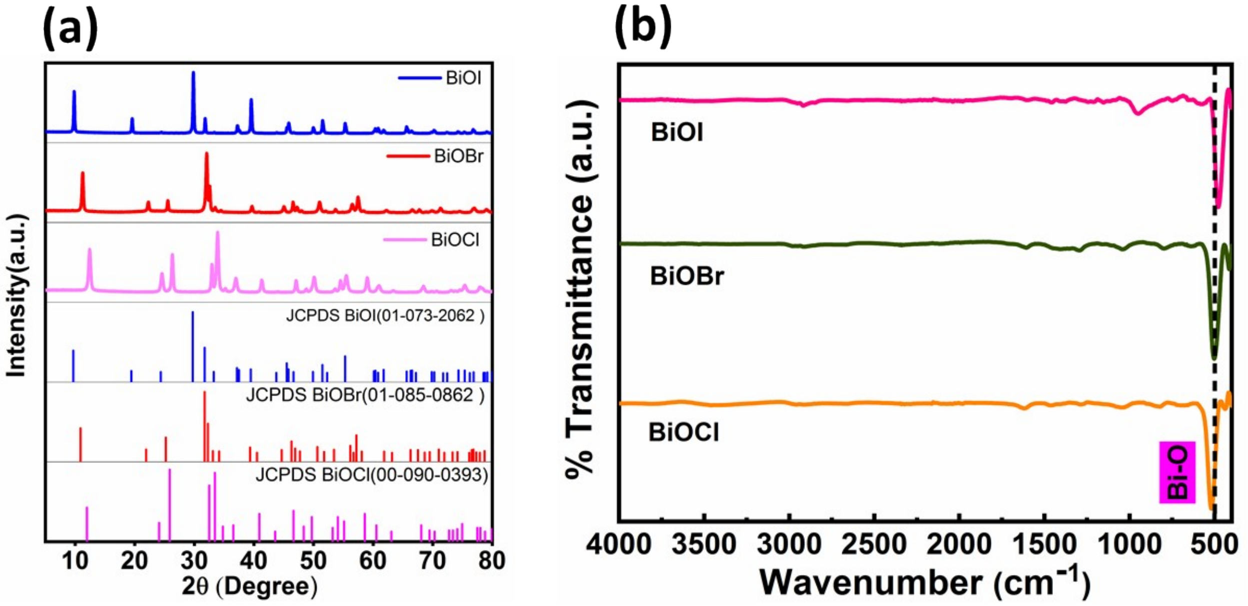

Fig. S1 (a) XRD pattern and (b) FTIR spectra of as synthesized BiOX.

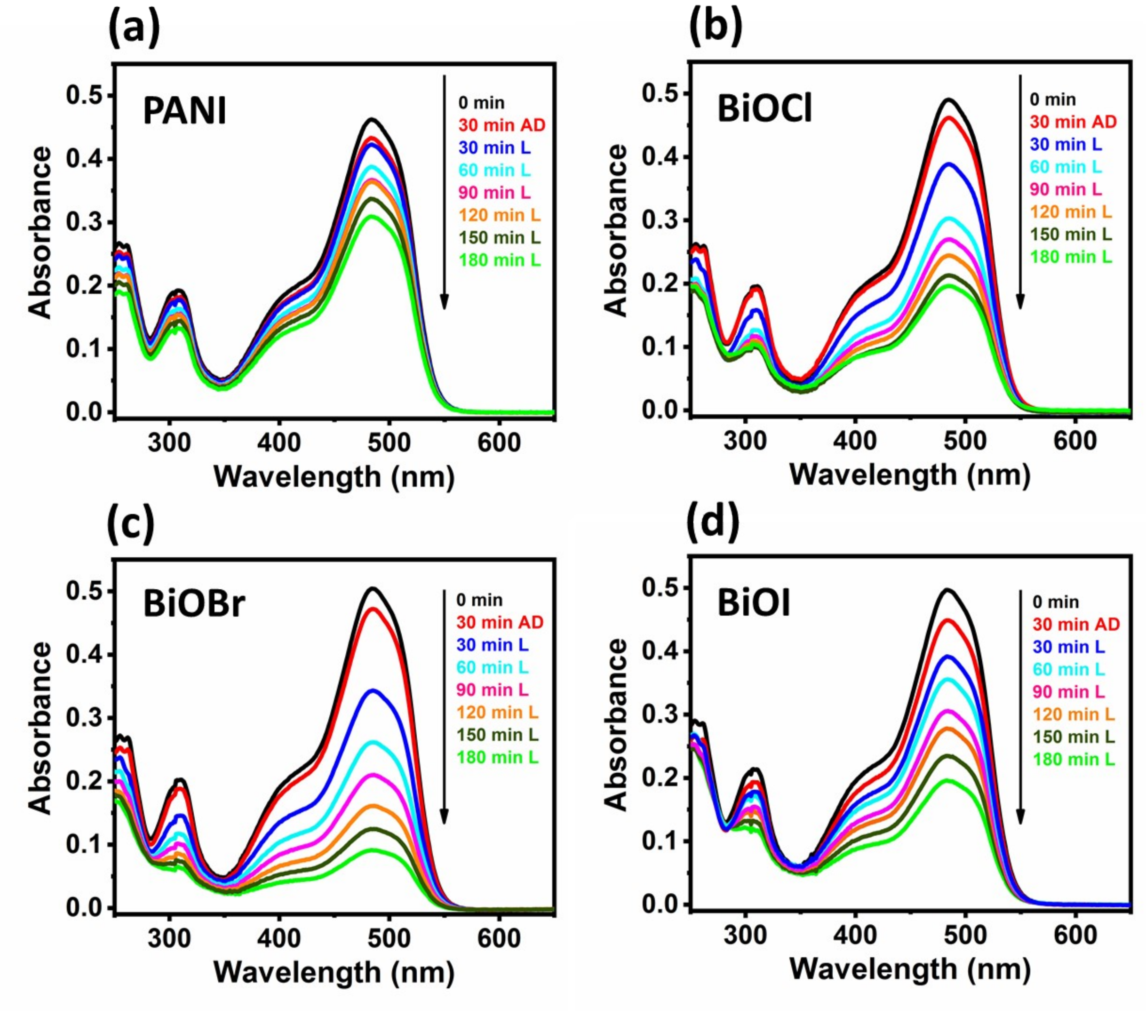

Fig. S2 UV-vis absorbance spectra of photocatalytic OII dye degradation under visible light for : (a) PANI, (b) BiOCl, (c) BiOBr, and (d) BiOI photocatalyst.

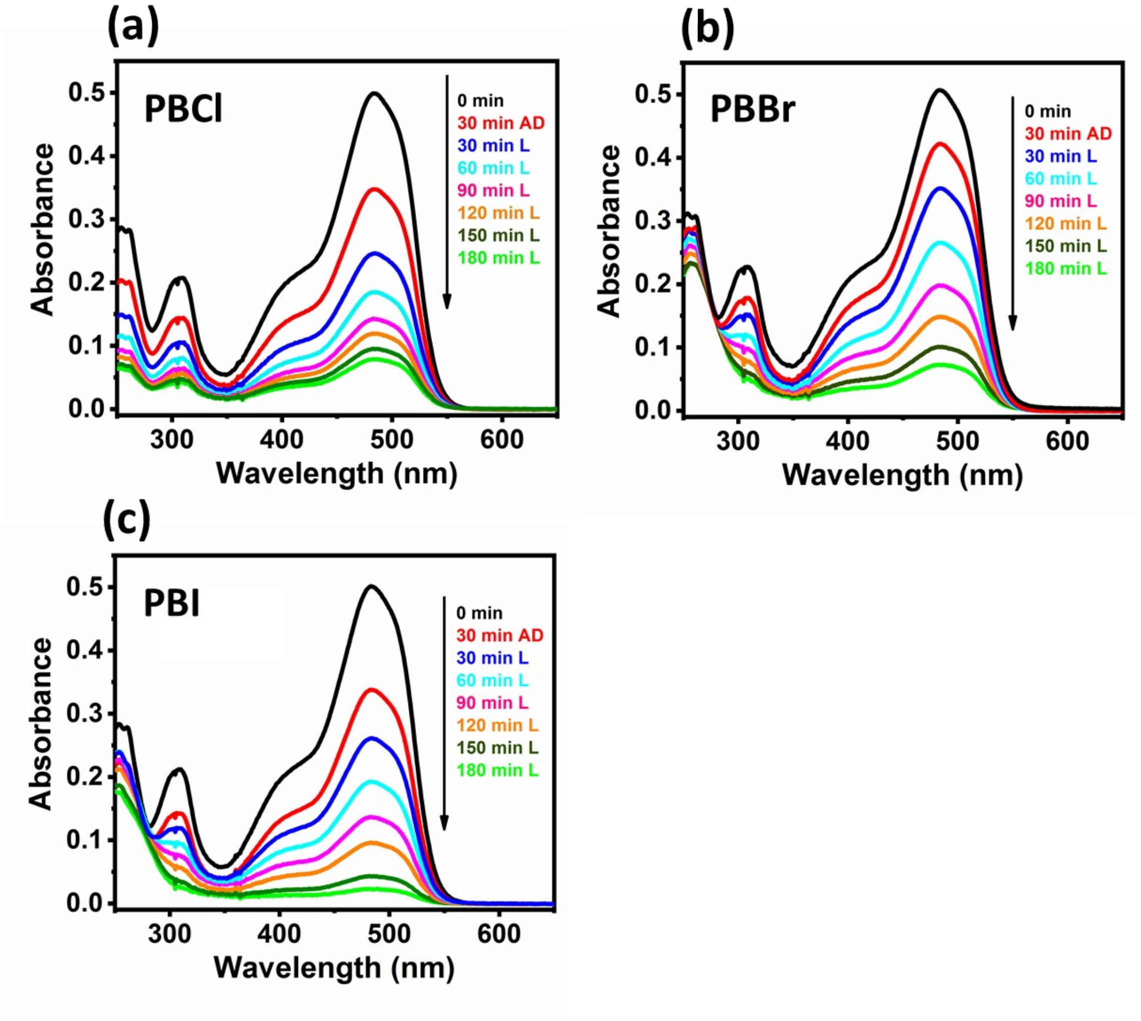

Fig. S3 UV-vis absorbance spectra of photocatalytic OII dye degradation under visible light for : (a) PBCl, (b) PBBr, and (c) PBI composite photocatalyst.

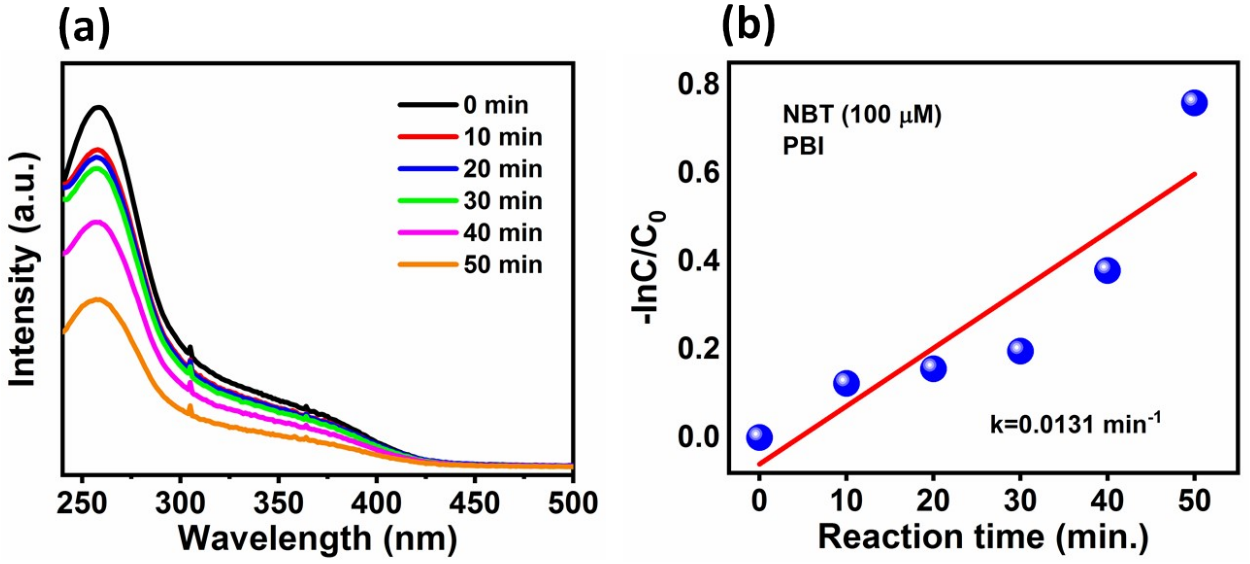

Fig. S4 (a) Time-resolved UV-visible absorbance spectra of NBT during visible light irradiation in presence of PBI composite and (b) the respective kinetic curve.

Concentration of $\bullet O_2^-$ radicals was calculated using the formula:

Concentration of $\bullet O_2^-$ radical = $\mathbf{k_{NBT} \times t \times C_{initial} \times 4}$

k_{NBT} = 0.0131min^{-1} (loss kinetic constant of NBT)

t = 50 min (the light exposure time)

$C_{initial}$ = 100 μM (NBT initial concentration)

4 is the constant coefficient between the reaction of NBT and $\bullet O_2^-$

Concentration of $\bullet O_2^-$ radical = 2.62×10^{-5} M

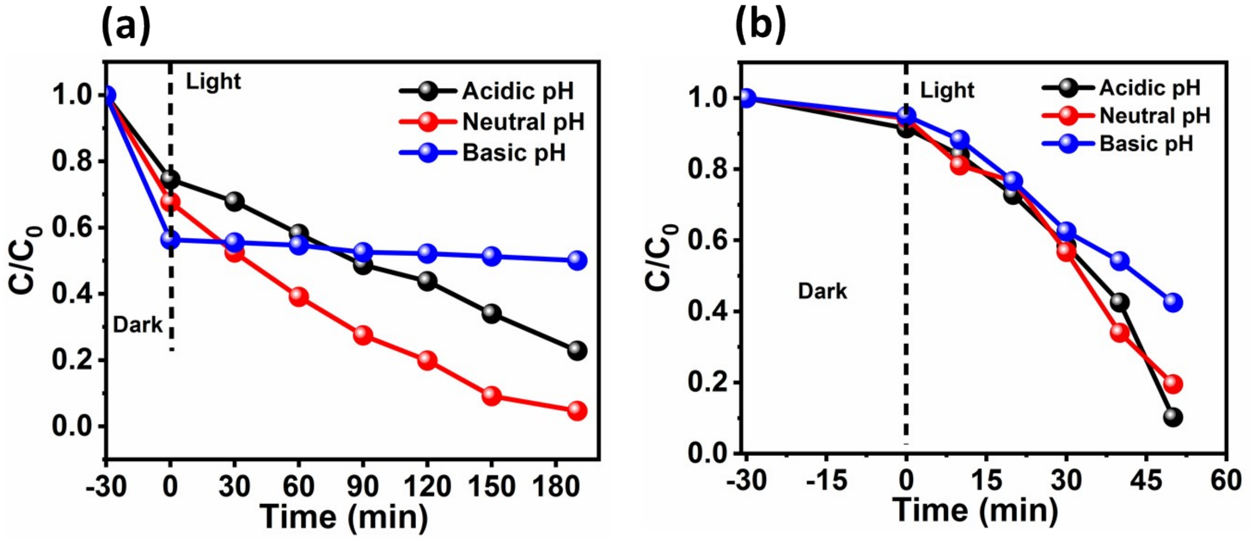

Fig. S5 Effect of varying pH (acidic, 0.1 M HCl neutral and basic, 0.1 M NaOH) on the photocatalytic performance of PBI on **(a)** degradation of OII dye, and **(b)** photocatalytic reduction of Cr(VI) to Cr(III).

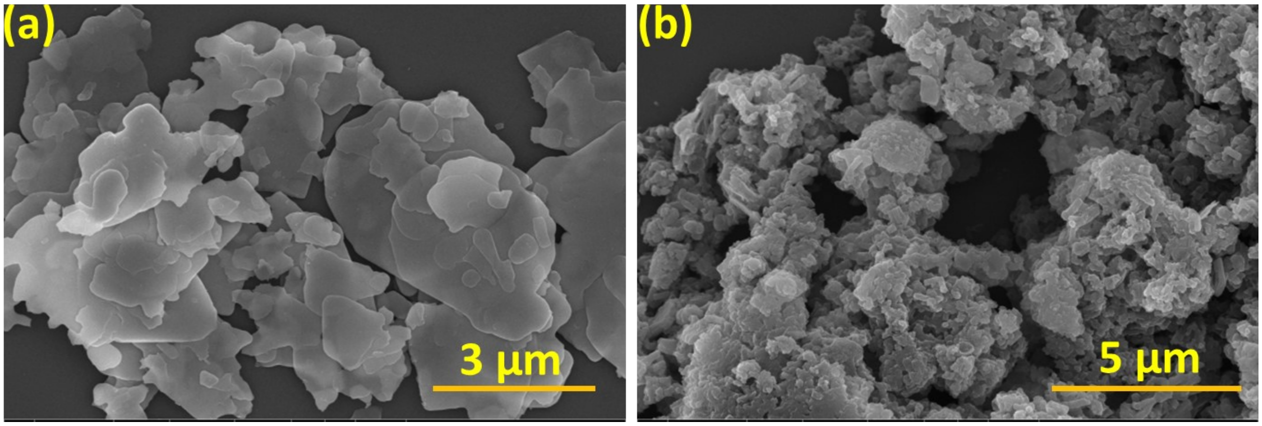

Fig. S6 FESEM images of (a) BiOI and (b) PANI

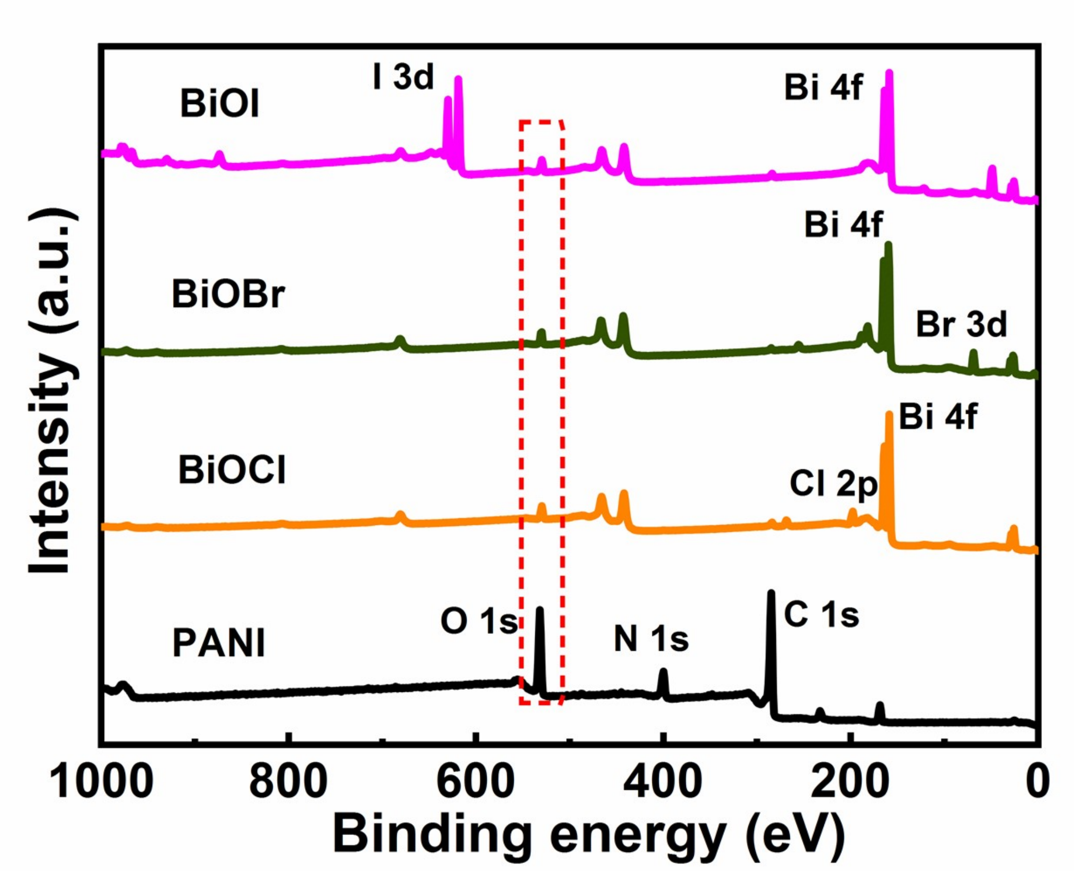

Fig. S7 XPS survey spectrum of PANI and as synthesized nanocomposites

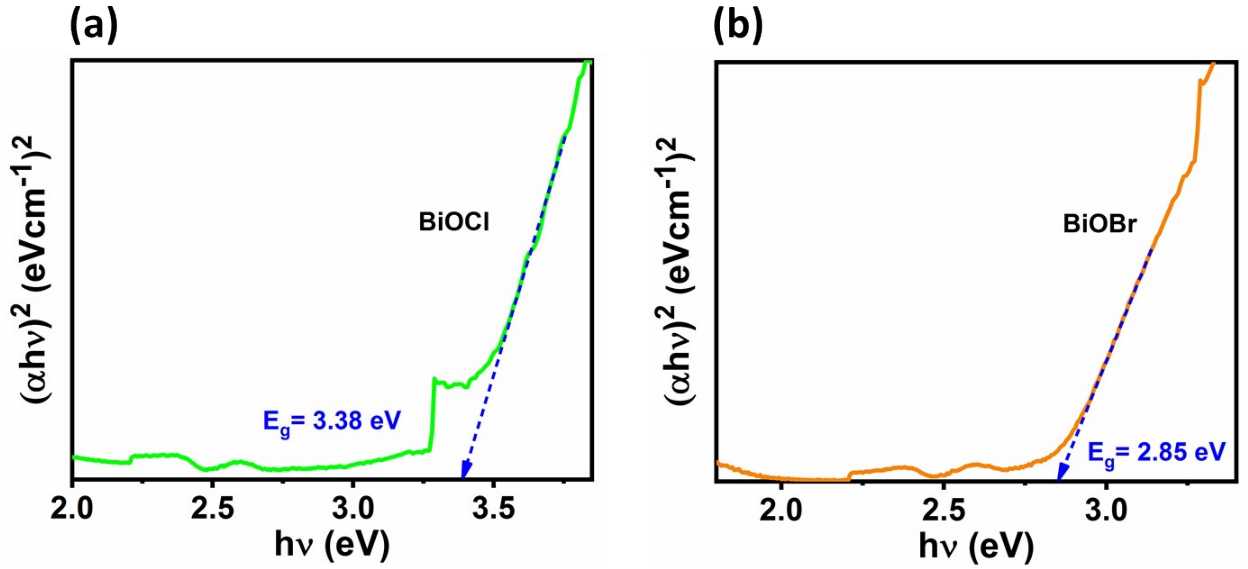

Fig. S8 Tauc plot showing band gap enegy of (a) BiOCl and (b) PANI BiOBr.

Table: S1 Comparison of the photocatalytic activity of the PANI-BiOI with some other hybrid composites for the degradation of various pollutants under visible light.

Photocatalyst	Pollutant, Conc. (ppm)	Catalyst amount (mg/mL)	Light source	Removal (%)	Time (min)	Ref.
ZnO/BiOI	MO, 10	1	Halogen tungsten lamp (500 W)	78	240	[1]
BiOI/Bi_2MoO_6	RhB, 7	1	Xe lamp (300 W)	95	90	[2]
2D/3D g-C_3N_4/BiOI	AR, 10	0.1	Mercury lamp (250 W)	74.64	150	[3]
Ppy-BiOI	RhB, 4<br>BPA, 30<br>2,4-DCP, 30<br>TC, 30	1<br>1<br>1<br>1	Xe lamp	83<br>50<br>29<br>61	300<br>300<br>300<br>300	[4]
AgI/BiOI	MO, 20<br>Phenol, 20	1<br>1	Xe lamp (300 W)	90.2<br>41.6	180<br>180	[5]
BiOI/$BiVO_4$	RhB, 5	0.6	Xe lamp (300 W)	97	75	[6]
NiS/BiOI	RhB, 20<br>TC, 50<br>Cr(VI), 50	0.3<br>0.3<br>0.27	Xe lamp (300 W)	99.8<br>96.3<br>92.8	200<br>140<br>180	[7]
Fe-nPPy/BiOI	CV, 10<br>TC, 20	0.5<br>1	White LED (192 W)	84<br>74	120<br>120	[8]
**PANI/BiOI**	**OII, 20**<br>**Cr(VI)**	**0.5**<br>**0.5**	**White LED (160 W)**	**95.3**<br>**90**	**180**<br>**50**	**Current work**

References:

1 J. Jiang, X. Zhang, P. Sun and L. Zhang, 2011, 20555–20564.

2 Y. Wang, M. Wang, J. Liu, L. Wang, H. Pang, Y. Su, J. Pan, Z. Z. Xue and Y. Peng, *Inorg. Chem.*, 2023, **62**, 9158–9167.

3 J. Li, L. Guan, L. Jiang, M. Xu, J. Li, J. Zuo, C. Tan and Z. Xia, *ACS Omega*, 2024, **9**, 361–370.

4 J. Xu, Y. Hu, C. Zeng, Y. Zhang and H. Huang, *J. Colloid Interface Sci.*, 2017, **505**, 719–727.

5 H. Cheng, B. Huang, Y. Dai, X. Qin and X. Zhang, *Langmuir*, 2010, **26**, 6618–6624.

6 S. Ni, T. Zhou, H. Zhang, Y. Cao and P. Yang, *ACS Appl. Nano Mater.*, 2018, **1**, 5128–5141.

7 C. T. Haile, N. Ahmad, C. W. Chiu and C. F. Jeffrey Kuo, *Chemosphere*, 2023, **323**, 138108.

8 R. Kumar, R. Gogoi, K. Sharma, A. Singh and P. F. Siril, *Environ. Sci. Adv.*, 2023, **3**, 85–96.
